# New MoDC-Targeting TNF Fusion Proteins Enhance Cyclic Di-GMP Vaccine Adjuvanticity in Middle-Aged and Aged Mice

**DOI:** 10.3389/fimmu.2020.01674

**Published:** 2020-08-07

**Authors:** Himanshu Gogoi, Samira Mansouri, Divya S. Katikaneni, Lei Jin

**Affiliations:** Division of Pulmonary, Critical Care and Sleep Medicine, Department of Medicine, University of Florida, Gainesville, FL, United States

**Keywords:** 3′, 5′-cyclic diguanylic acid (cyclic di-GMP), vaccine, aging, monocyte-derived dendritic cells (moDCs), tumor necrosis factor (TNF)

## Abstract

Cyclic dinucleotides (CDNs) are promising vaccine adjuvants inducing balanced, potent humoral, and cellular immune responses. How aging influences CDN efficacy is unclear. We examined the vaccine efficacy of 3′,5′-cyclic diguanylic acid (cyclic di-GMP, CDG), the founding member of CDNs, in 1-year-old (middle-aged) and 2-year-old (aged) C57BL/6J mice. We found that 1- and 2-year-old C57BL/6J mice are defective in CDG-induced memory T helper (Th)1 and Th17 responses and high-affinity serum immunoglobulin (Ig)G, mucosal IgA production. Next, we generated two novel tumor necrosis factor (TNF) fusion proteins that target soluble TNF (solTNF) and transmembrane TNF (tmTNF) to monocyte-derived dendritic cells (moDCs) to enhance CDG vaccine efficacy in 1- and 2-year-old mice. The moDC-targeting TNF fusion proteins restored CDG-induced memory Th1, Th17, and high-affinity IgG, IgA responses in the 1- and 2-year-old mice. Together, the data suggested that aging negatively impacts CDG vaccine adjuvanticity. MoDC-targeting TNF fusion proteins enhanced CDG adjuvanticity in the aging mice.

## Introduction

The current COVID-19 pandemic has highlighted the vulnerability of aging populations to emerging pathogens where people 45 and older account for ~97% of COVID-19 deaths (1). Vaccination offers the most efficient and cost-effective method to stop infectious diseases. However, vaccine efficacy is substantially reduced with age due to the progressive age-related decline of innate and adaptive immune responses (immunosenescence) (2–4). Immunosenescence also posts a significant challenge for the development of immunotherapeutic agents. The number of Americans ages 65 and older will double and reach 80 million in 2040 (5). Improving the efficacy of vaccines and immunotherapeutic agents in the aging population is critical to public health.

The biggest challenge in vaccination response in the aged is the inadequate antibody response (6, 7). Nevertheless, improving vaccine efficacy in the aged is achievable. For instance, while the efficacy of Prevnar13^®^ decreases with age (8) and is only ~45% effective in adults 65 years or older (9), the Shingrix vaccine for shingles is 90% effective in people over 70. The underlying mechanism behind the successful Shingrix in the aged, however, is unclear. Current vaccine strategies for the elderly are empirical. They include higher antigen dose, adjuvanted vaccine, and alternative route of immunization (10–12). We reasoned that mechanism-guided vaccine design could aid in the development of effective vaccines for the aged.

Cyclic dinucleotides (CDNs) are a promising class of vaccine adjuvants inducing potent, balanced, and long-lasting humoral and cellular adjuvant responses for cancers and infectious diseases (13–15). CDNs include the bacterial second messenger, 3′,5′-cyclic diguanylic acid (cyclic di-GMP, CDG), cyclic di-AMP, mammalian second messenger 2′3′-cyclic GMP-AMP, and synthetic RpRp-ssCDA (15). Multiple clinical trials are ongoing to examine CDN efficacy in cancer immunotherapy (ClinicalTrials.gov NCT02675439, NCT03010176, NCT03172936, NCT03937141, and NCT0414414). Almost all studies on CDN adjuvanticity were done in young mice (<3 months old). How aging influences CDN efficacy *in vivo* is not clear.

CDG was the founding member of CDNs and showed excellent vaccine efficacy in infectious diseases and immunotherapy (16–18). Mechanistically, CDG adjuvanticity depends on stimulator of interferon genes (STING)-induced tumor necrosis factor (TNF) production *in vivo* (19, 20) and is mediated by DCs (21). DCs are developmentally and functionally heterogeneous populations. We recently showed that CDG adjuvanticity is mediated by conventional DC2 (cDC2) and monocyte-derived DCs (moDCs) *in vivo* (22).

In this study, we examined CDG adjuvanticity in middle-aged and aged C57BL/6J mice. We further developed a mechanism-guided moDC-targeting strategy to enhance CDG adjuvanticity in the middle-aged and aged mice.

## Methods

### Key Resources Table

**Table:** 

**REAGENT or RESOURCE**	**SOURCE**	**IDENTIFIER**
**Antibodies**		
Anti-mouse CD4-PE/Cy7 (clone: GK1.5)	BioLegend	Cat#100422
Anti-mouse CD45-PercP/Cy5.5 (clone: 30-F11)	Biolegend	Cat#103131
Anti-mouse MHCII(I-A/I-E)-Brilliant Violet 421 (clone: M5/114.15.2)	BioLegend	Cat#107636
Anti-mouse CD11c-APC/Cy7 (clone: N418)	Bi olegend	Cat#117323
Anti-mouse/human CD11b-PE/Cy7 (clone: M1/70)	BioLegend	Cat#101216
Anti-mouse CD64-PerCP/Cy5.5 (clone: X54-5/7.1)	BioLegend	Cat#139307
Anti-mouse TNFR2-APC (Clone: REA228)	Miltenyi Biotec	Cat#130-104-698
Anti-mouse PD1–FITC (Clone: 29F.1A12)	BioLegend	Cat#135214
Anti-mouse/human pRelB-PE (clone: D41B9)	Cell Signaling Technology	Cat#:13567
Anti-mouse CXCR5–APC (clone: L138D7)	BioLegend	Cat#145506
Anti-mouse CD19–PerCP/Cy5.5 (clone: 1D3/CD19)	BioLegend	Cat#152405
Anti-mouse CD86–APC-Cy7 (clone: GL-1)	BioLegend	Cat#105029
Anti-mouse IgG2c-HRP	Southern Biotech	Cat#1078-05
Anti-mouse IgG1-HRP	Southern Biotech	Cat#1071-05
Anti-mouse IgG-HRP	Southern Biotech	Cat#1033-05
Anti-mouse IgA-HRP	Southern Biotech	Cat#1040-05
**Chemicals, Antigen, and ELISA kit**		
H1N1-nucleoprotein	SinoBiological	cat#11675-V08B
OVA	Invivogen	Cat#vac-pova
Cyclic di-GMP (vaccine-grade)	Invivogen	Cat# vac-nacdg
NP_23_-BSA	Biosearch Technologies	Cat# N-5050H
Tissue protein extraction reagent (T-PER)	ThermoFisher	Cat#78510
ACK lysis buffer	Thermofisher	Cat# A1049201
NP_2_-BSA	Biosearch Technologies	Cat# N-5050XL
Protease inhibitors	Roche,	Cat#11836153001
NP_6_CGG	Biosearch Technologies	Cat# N-5055A
TNF_D221N/A223R_-Fc (mouse IgG2A) fusion protein	Creative^®^ Biolabs	Custom made
TNF-Fc (mouse IgG2A) fusion protein	Creative^®^ Biolabs	Custom made
Mouse TNF ELISA kit	ThermoFisher	Cat # 88-7324-22
Mouse IFNγ ELISA kit	ThermoFisher	Cat # 88-7314-22
Mouse IL-17a ELISA kit	ThermoFisher	Cat # 88-8711-22
Mouse IL-13 ELISA kit	ThermoFisher	Cat # 88-7137-22
**Experimental Models: Organisms/Strains**		
Mouse: CD11c^Cre^	Jackson Laboratory	Cat#008068
Mouse: IRF4^fl/fl^	Jackson Laboratory	Cat#009380
C57BL/6J	Jackson Laboratory	Cat#000664
**Software and Algorithms**		
FlowJo version 10.1r1	FlowJo	http://www.flowjo.com
Prism6	GraphPad	http://www.graphpad.com

### Mice

All mouse experiments were performed by the regulations and approval of the Institutional Animal Care and Use Committee from the University of Florida (IACUC no: 201909362). C57BL/6J mice were obtained, bred, maintained, and aged in the Animal Research Facility at the University of Florida. Both males and females were used to carry out the experiments.

### Immunization and Sample Collection

For intranasal immunization, mice were immunized with protein antigen H1N1-nucleoprotein (NP), pneumococcal surface protein A (PspA), 4-hydroxy-3-nitrophenylacetyl hapten conjugated with chicken gamma globulin (NP_6_CGG) (1.5 μg/mouse) alone or mixed with CDG (5 μg/mouse) and/or soluble TNF (solTNF)-Fc [immunoglobulin G2A (IgG2A)] (100 ng/mouse) and transmembrane TNF (tmTNF)-Fc (IgG2A) (100 ng/mouse) in total 40 μl endotoxin-free phosphate buffered saline (PBS). Sera were collected after 30 and 60 days of the last immunization. Bronchoalveolar lavage fluid (BALF) was collected on day 60.

The TNF-Fc (IgG2A) (lot # CB181108) and TNF_D221N/A223R_-Fc (IgG2A) (lot # CB190829) fusion proteins were made by Creative^®^ Biolabs. The fusion protein has N-Terminal Histidine tag. They were expressed in CHO cells and purified with affinity chromatography. The purity is >95% as determined by sodium dodecyl sulfate (SDS)-polyacrylamide gel electrophoresis (PAGE) (Coomassie blue densitometry). Endotoxin level of the fusion proteins is less than 0.5 EU/mg fusion protein.

The following reagent was obtained through BEI Resources, National Institute of Allergy and Infectious Diseases (NIAID), National Institutes of Health (NIH): *Streptococcus pneumoniae* Family 2, Clade 3 Pneumococcal Surface Protein A (PspA UAB099) with C-Terminal Histidine Tag, Recombinant from *Escherichia coli*, NR-33179.

### Antibody ELISA

A 96-well, flat-bottomed polystyrene plate was coated with 500 ng of H1N1-NP, PspA, or NP_2_BSA, NP_23_BSA, washed with 1 × PBS with Tween 20 (PBST), blocked with 2% bovine serum albumin (BSA) in 1 × PBS, and incubated at 37°C (20). After wash, serum samples were serially diluted in PBS, added to the wells in triplicate, and incubated at 37°C for 1 h. The plate was washed. Horseradish peroxidase (HRP)-conjugated anti-mouse IgG/IgG1/IgG2c/IgA was added at a dilution of 1:5,000 and incubated at 37°C for 1 h. After washing, 3,3′,5,5′-tetramethylbenzidine (TMB) substrate reagent was added and incubated for 20 min in the dark. The reaction was stopped using 50 μl of stop solution, and absorbance reading noted at 450 nm.

### *Ex-vivo* Recall of Lung Cells and Splenocytes

Lung and spleen cells were harvested and digested as before (21). The cells were resuspended in RPMI media containing 10% fetal bovine serum (FBS) in a 96-well tissue culture plate at a concentration of 1 × 10^6^/well. The cells were stimulated with 1 μg H1N1-NP or 2 μg NP_6_CGG and incubated for 4 days at 37°C and 5% CO_2_. Culture supernatants were collected, and cytokine levels were evaluated for interleukin (IL)-13, IL-5, interferon (IFN)-γ, and IL-17a by ELISA.

### Flow Cytometry

Lungs were harvested and processed as discussed above. The single-cell suspension was prepared and analyzed by BD™ LSR II and FACScan flow cytometry (21).

### Gating Strategy for Lung Dendritic Cells

cDC1 are MHCII^hi^CD11c^+^CD11b^−^CD64^−^, moDCs are MHCII^hi^CD11c^+^CD11b^+^CD64^+^, and cDC2 are MHCII^hi^CD11c^+^CD11b^+^CD64^−^ (21). Antibody stain was performed at 4°C for 20 min. Please see the *Key Resources* section above for a detailed list of antibodies used.

### Detection of Lung Tumor Necrosis Factor

Mice were intranasally immunized with either PBS or CDG (5 μg) and sacrificed after 5 h by CO_2_ asphyxiation. Lungs were perfused with ice-cold PBS, removed and stored in 0.7 ml Tissue Protein Extraction Reagent (T-PER) containing protease inhibitors at −80°C. Later, the lung was thawed on ice and homogenized with Minilys^®^ (Precellys, 5,000 RPM for 30 s) using Precellys lysing kit. Lung homogenates were transferred to a 1.5-ml tube and harvested at 14,000 g for 30 min at 4°C. The supernatant was collected and analyzed for TNF production by ELISA.

### Statistical Analysis

GraphPad Prism v6.05 software was used for data preparation and statistical analysis. One-way or two-way ANOVA followed by Tukey's multiple comparison tests were employed. *p* < 0.05 was considered significant. ^*^ < 0.05, ^**^ < 0.01, ^***^ < 0.001, ^****^ < 0.0001. n.s. represents non-significant.

### Experimental Design

Data exclusion was justified when positive or negative control did not work. All experiments will be repeated at least three times. All repeats are biological replications that involve the same experimental procedures on different mice. Experiments comparing different genotypes, adjuvant responses are designed with individual treatments being assigned randomly. Where possible, treatments will be assigned blindly to the experimenter by another individual in the lab. When comparing samples from different groups, samples from each group will be analyzed in concert, thereby preventing any biases that may arise from analyzing individual treatments on different days.

## Results

### 3′,5′-Cyclic Diguanylic Acid Adjuvant Activity Is Impaired in Aged Mice

**Figure 1:**
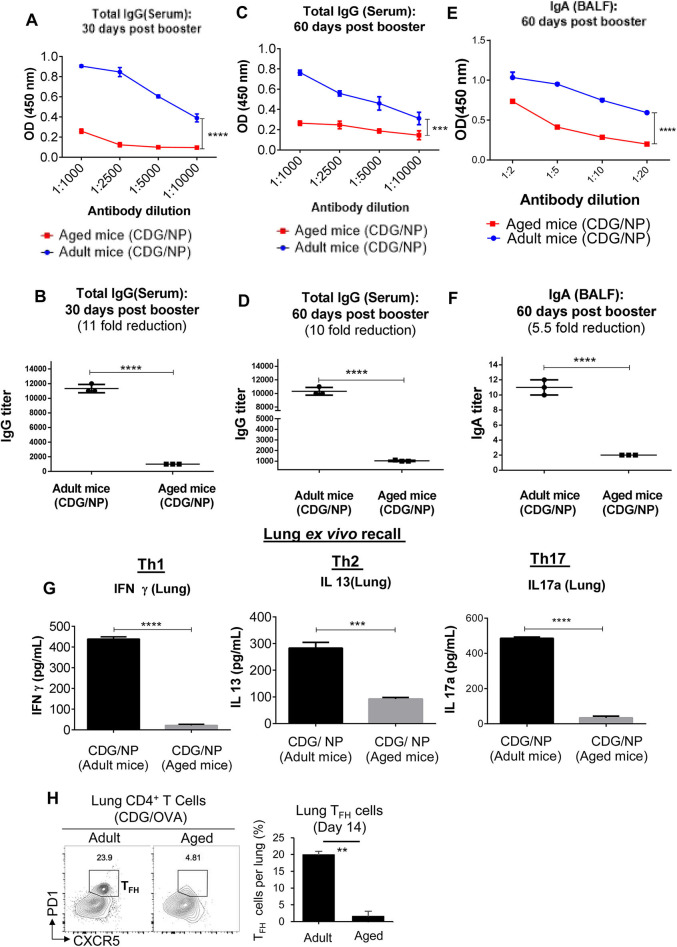
3′,5′-Cyclic diguanylic acid (cyclic di-GMP, CDG) adjuvanticity is decreased in aged mice. **(A–F)** Adult (~3-month-old) and aged (~18-month-old) C57BL/6 wild-type (WT) mice (*n* = 4 mice/group) were immunized intranasally (i.n.) with CDG (5 μg) plus influenza nucleoprotein (NP) (1.5 μg) on days 0 and 14. Antibody titers were determined from serum (day 30 and 60) and bronchoalveolar lavage fluid (BALF) samples 60 days post the last immunization. Immunoglobulin (Ig)G and IgA endpoint titers were determined by comparing the OD450 from adult immunized mice with aged immunized mice. Data are representative of three independent experiments. **(G)** Lung cells from the immunized mice in **(A)** were recalled with NP (1 μg) *ex vivo* conditions for 4 days. Th17, Th1, and Th2 cytokines were measured in the culture supernatant. Data are representative of three independent experiments. **(H)** Flow cytometry analysis of T follicular helper (TFH) formation in lung cells of adult and aged C57BL/6 mice on day 14 post CDG (5 μg)/ovalbumin (OVA) (1.5 μg) immunization (i.n.). (*n* = 4 mice/group). Data are representative of three independent experiments. Significance value, *p*, for **(A–F)** was calculated between adult and aged mice groups using two-way ANOVA followed by Tukey's multiple comparison test **(A,C,E)** and Student's *t-*test **(B,D,F)**. Significance value, *p*, for **(G,H)** was calculated between adult and aged mice groups using one-way ANOVA followed by Tukey's multiple comparison test. Error bars represent mean ± SEM. Statistical significance is represented by *p*; ^**^p < 0.01, ^***^p < 0.001, ^****^p < 0.0001.

CDG induces balanced, potent, and long-lasting vaccine adjuvant responses in young animals (16, 20–22). To investigate the influence of aging, we compared CDG adjuvanticity in 18-month-old aged and 3-month-old adult C57BL/6J mice. Mice were immunized [intranasally (i.n.)] with CDG/H1N1- NP twice at a 2-week interval. Serum anti-H1N1-NP antibody titer was determined on days 30 and 60 after the last immunization. Unexpectedly, aged mice showed significantly (~10-folds) reduction of anti-H1N1-NP IgG titer (*p* < 0.0001; *p* < 0.001) than the adult mice (Figures 1A–F). Similarly, on day 60, IgA antibody titer in BALF was significantly lower (~5-folds, *p* < 0.0001) in aged mice than the adult mice.

CDG immunization generates potent and long-lasting memory T helper (Th)1, Th2, Th17 response in adult mice (16, 20–22). However, *ex vivo* recall assay showed that 60 days post-immunization, aged mice had dramatically (10-folds) reduced memory Th1 and Th17 responses in the lung (Figure 1G). The lung memory Th2 responses were also reduced by half (Figure 1G). CDG induced lung T follicular helper (TFH), which is critical for its adjuvant responses (22). We examined lung TFH cells on day 14 post-immunization. Lungs from immunized aged mice showed a 10-fold reduction of CXCR5^+^ PD1^+^ CD4^+^ TFH cells than the adult mice (Figure 1H). Together, the data indicated that CDG adjuvanticity was impaired in the aged mice.

### 3′,5′-Cyclic Diguanylic Acid Did Not Activate Lung TNFR2^+^ cDC2 or Monocyte-Derived Dendritic Cells in Aged Mice

**Figure 2:**
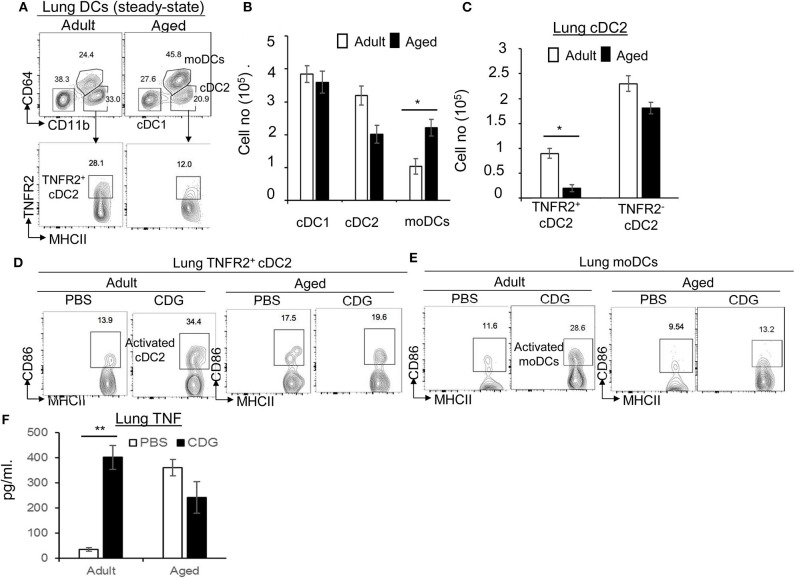
3′,5′-Cyclic diguanylic acid (cyclic di-GMP, CDG) mediated lung TNFR2^+^ cDC2, or monocyte-derived dendritic cell (moDC) activation is impaired in aged mice. **(A–C)** Flow cytometry analysis and quantification of lung DC subsets of adult and aged C57BL/6 mice at steady state. *n* = 4 mice/group. Data are representative of three independent experiments. **(D,E)** Flow cytometry analysis of CD86 expression in lung TNFR2^+^ cDC2 and moDC of adult and aged C57BL/6 mice treated intranasally (i.n.) with PBS or CDG (5 μg) for 16 h. *n* = 4 mice/group. Data are representative of three independent experiments. **(F)** Quantification of TNF production in the lung homogenates of adult and aged mice treated (i.n.) with PBS or CDG (5 μg) for 5 h. *n* = 4 mice/group. Data are representative of three independent experiments. Significance value, *p*, was calculated using one-way ANOVA followed by Tukey's multiple comparison test. Error bars represent mean ± SEM. Statistical significance is represented by *p*; ^*^p < 0.05, ^**^p < 0.01.

CDG activates lung DCs to mediate its adjuvanticity *in vivo* (21). The lung DCs are functionally heterogeneous. In particular, lung cDC2 and moDCs play a pivotal role in the CDG vaccine adjuvanticity (22). We found that aged mice had increased moDCs in the lung (Figures 2A,B). On the other hand, lung cDC2 was decreased in the aged mice, but the difference was not significant (Figures 2A,B). cDC2 consists of functionally distinct TNFR2^+^ and TNFR2^−^ cDC2 subsets (22). The TNFR2^+^ cDC2 subset (R2D2) mediates the cellular immune responses of CDG adjuvant, while the TNFR2^−^ cDC2 subset activates moDCs to mediate the humoral responses of CDG (22). Interestingly, lung R2D2 population was significantly decreased in the aged mice (Figure 2C).

Next, we examined CDG-induced lung DC activation in the aged mice. Intranasal administration of CDG did not activate lung R2D2 or moDCs in the aged mice, as indicated by the lack of CD86 upregulation (Figures 2D,E). CDG activates lung cDC2 to produce lung TNF (Figure 2F) that is critical for CDG adjuvanticity (19, 20, 22). We measured lung TNF in aged and adult mice. We first noticed that aged mice had elevated lung TNF levels compared to adult mice at steady state (Figure 2F). Second, CDG did not increase lung TNF production in the aged mice (Figure 2F), suggesting that lung cDC2 from the aged mice is defective in response to CDG.

Last, TNFR2 expression on moDCs is essential for CDG adjuvanticity *in vivo* (22). Consistently, CDG induced less lung TNFR2^+^ moDCs in the aged mice than the adult mice (Figure S1A). TNFR2 signals *via* the non-canonical nuclear factor (NF)-κB pathway RelB. Again, CDG-induced pRelB was reduced in lung moDCs from the aged mice (Figure S1B). Together, the data suggested that CDG-induced lung R2D2 or moDC activation was impaired in the aged mice.

### 3′,5′-Cyclic Diguanylic Acid-Induced Th1 and Th17 Responses Were Impaired in the 1-Year-Old Middle-Aged Mice

Adult mice (~3 months old), not aged mice (~18 months old), exhibit potent CDG-induced humoral and cellular responses (Figure 1). We asked at what age CDG adjuvant responses start to wane. We examined the 1-year-old mice, which are the equivalent of ~42.5-year-old middle-aged humans. We immunized 1-year-old mice with CDG/NP_6_CGG twice at 2 weeks' interval and examined antibody and memory Th responses after 60 days. We chose antigen NP_6_CGG so that we can separate vaccine-induced low-affinity (anti-NP_23_) and high-affinity (anti-NP_2_) antigen-specific antibodies influenced by aging. High-affinity antibody formation is a hallmark of germinal center formation and provides rapid neutralization of extracellular pathogens.

**Figure 3:**
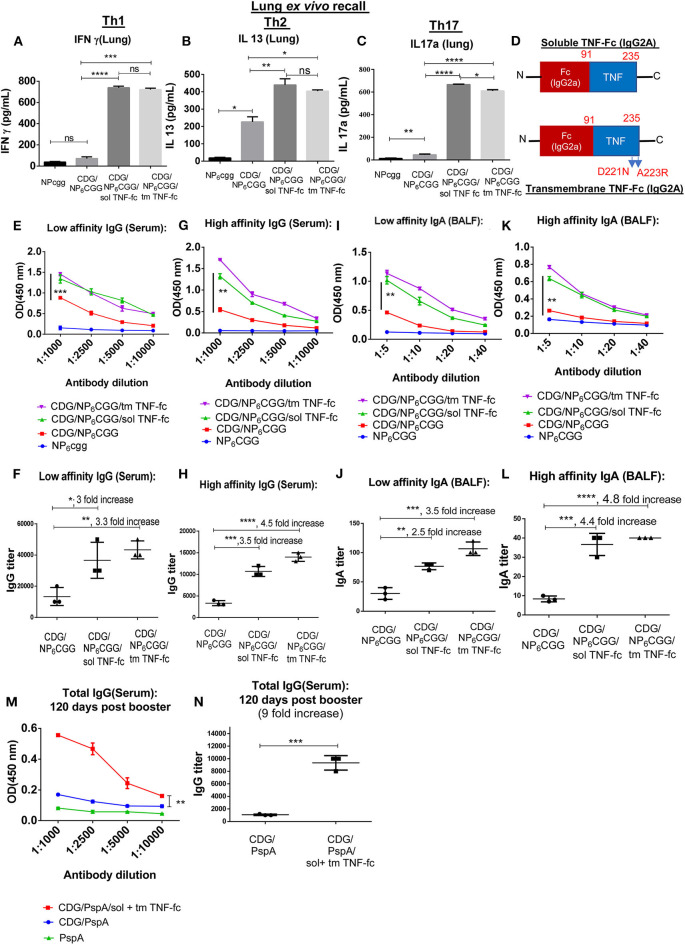
SolTNF-Fc [immunoglobulin (Ig)G2A] and tmTNF-Fc (IgG2A) enhanced 3′,5′-cyclic diguanylic acid (cyclic di-GMP, CDG)-induced lung memory Th and antibody response in 1-year-old mice. One-year-old C57BL/6 mice were immunized intranasally (i.n.) twice with NP_6_CGG, CDG (5 μg)/NP_6_CGG (1.5 μg), CDG/NP_6_CGG/solTNF-Fc (100 ng), or CDG/NP_6_CGG/tmTNF-Fc (100 ng) on days 0 and 14. *n* = 4 mice/group. Data are representative of three independent experiments. **(A–C)** Sixty days post the last immunization, lung cells from immunized mice were recalled *ex vivo* with NP_6_CGG (2 μg) for 4 days. Th1, Th2, and Th17 cytokines were determined in the culture supernatant by ELISA. Significance value was calculated by one-way ANOVA followed by Tukey's multiple comparison test. Error bar represents mean ± SEM. ^**^p < 0.01, ^***^p < 0.001. **(D)** A cartoon of the moDC-targeting TNF fusion proteins. **(E–L)** Serum and bronchoalveolar lavage fluid (BALF) samples were collected from mice in **(A)** and analyzed for NP-specific antibody titers by ELISA. IgG and IgA endpoint titers were determined by comparing the OD450 from mice immunized with NP_6_CGG. Significance value was calculated by two- way ANOVA followed by Tukey's multiple comparison test. Error bar represents mean ± SEM. ^*^p < 0.05, ^**^p < 0.01, ^***^p < 0.001, ^****^p < 0.0001, n.s., not significant. **(M,N)** One-year-old C57BL/6 mice were immunized (i.n.) twice with PspA, CDG/PspA, or CDG/PspA/(solTNF-Fc + tmTNF-Fc) on days 0 and 14. Serum samples were collected 120 days post last immunization, and anti-PspA specific antibody titer was determined by ELISA **(M)**. Antibody titer was quantified by comparing the OD450 of immunized mice with PspA only immunized mice **(N)**. *n* = 4 mice/group. Data are representative of three independent experiments. Significance value was calculated by two-way ANOVA followed by Tukey's multiple comparison test **(M)** or Student's *t*-test **(N)**. Error bar represents mean ± SEM. ^**^p < 0.01, ^***^p < 0.001.

Surprisingly, while CDG still induced antigen-specific Th2 responses in the lung of 1-year-old mice (Figure 3B; Figure S2A), CDG barely induced lung memory Th1 or Th17 responses in the 1-year-old mice (Figures 3A,C). Consistently, CDG induced antigen-specific IgG1, but not Th1-related IgG2C in the 1-year-old mice (Figures S2B,C). Next, we examined the high- and low-affinity antibodies in CDG-immunized 1-year-old mice. We found that CDG did not induce high-affinity lung IgA in the 1-year-old mice (Figures 3K,L). CDG still induced low-affinity lung IgA (Figures 3I,J). We reasoned that 1-year-old mice might be defective in CDG-induced antibody affinity maturation in the lung. In the serum, CDG induced low-affinity IgG, but the titers of high-affinity IgG was low (Figures 3E,F,G,H). The data, thus, suggested that CDG-induced memory Th1, Th17 responses, and high-affinity antibody production were impaired in the 1-year-old middle-aged mice.

### Design a Monocyte-Derived Dendritic Cell-Targeting TNF-Fusion Protein to Enhance 3′,5′-Cyclic Diguanylic Acid Adjuvant Responses in Aging Mice

The discovery that 1-year-old healthy mice already had waning CDG vaccine response was potentially significant in the translational research of CDNs. The median age for COVID-19 patients in the US is ~63 years old (23). The median age for pneumococcal meningitis patients is 41 years old (24). The median age for a recent CDN cancer clinical trial was 61 years old (ClinicalTrials.gov NCT02675439). Here, we discovered that CDG adjuvanticity depends on age. To translate CDG research into the clinic, we need methods to improve CDG adjuvanticity in aging humans.

MoDC activation is essential for CDG adjuvanticity (22). Aged mice had defective moDC activation, but their numbers increased (Figure 2). Meanwhile, lung TNF, including solTNF, and tmTNF, mediates CDG adjuvanticity by activating moDCs (20, 22). Last, aged mice had impaired CDG-induced lung TNF (Figure 2). We hypothesized that supplementing moDC-targeting solTNF and tmTNF might enhance CDG adjuvanticity in aging mice.

We generated TNF-Fc (IgG2A) and TNF_D221N/A223R_-Fc (IgG2A) fusion proteins to target solTNF or tmTNF to moDCs (Figure 3D). The TNF_D221N/A223R_ mutant mimics tmTNF that binds only to TNFR2, not TNFR1 (25). MoDCs express the high-affinity FcR, FcγRI (also known as CD64) that is not found on cDCs or lymphocytes (26). FcγRI bind the Fc of IgG2a with the highest affinity (10^8^ M^−1^), more than 1,000-fold higher than its next binding partner IgG2b-Fc (27). We showed that intranasal administration of allophycocyanin (APC)-conjugated mouse IgG2A was taken up exclusively by CD64^+^ lung cells, including MHC II^hi^ CD64^+^ moDCs (28). The CD64^+^ major histocompatibility complex (MHC) class II^low/int^ macrophages were also targeted (28). However, macrophages are dispensable for CDG mucosal adjuvanticity *in vivo* (21, 22).

To test the efficacy of moDC-targeting TNF fusion proteins, we used the IRF4^fl/fl^CD11c^cre^ mice. CDG does not generate lung TNF or vaccine responses in the IRF4^fl/fl^CD11c^cre^ (22), thus facilitated the TNF fusion proteins complement experiment. Furthermore, IRF4^fl/fl^CD11c^cre^ mice only have cDC1 and moDCs. cDC1 is dispensable for CDG adjuvanticity (22). If either solTNF-Fc or tmTNF-Fc restores antibody responses in the IRF4^fl/fl^CD11c^cre^ mice, it will strongly suggest that these TNF fusion proteins act through moDCs to enhance CDG adjuvanticity *in vivo*.

We found that intranasal administration of tmTNF-Fc (IgG2A) plus CDG generated high-affinity serum IgG antibody in the IRF4^fl/fl^CD11c^cre^ mice (Figure S3), suggesting that tmTNF-Fc (IgG2A) can enhance CDG-induced humoral response in the absence of cDC2. This is an important feature as we showed that aged mice had a decreased number of cDC2, especially R2D2 that is critical for CDG adjuvanticity. Thus, tmTNF-Fc (IgG2A) may be suitable to enhance CDG antibody responses in the aged mice where lung R2D2 is limited.

SolTNF-Fc (IgG2A) did not restore CDG-induced antibody responses in the IRF4^fl/fl^CD11c^cre^ mice (Figure S3). This was consistent with the previous finding that TNFR2 expression of moDCs is important for CDG-induced lung TFH cells and GC (Germinal Center) B cell production (22). Last, as expected, neither tmTNF-Fc (IgG2A) nor solTNF-Fc (IgG2A) restored the memory Th responses in the IRF4^fl/fl^CD11c^cre^ mice (28) as we previously demonstrated that R2D2 is required for CDG-induced cellular immunity (22).

### Monocyte-Derived Dendritic Cell-Targeting SolTNF and TmTNF Enhances 3′,5′-Cyclic Diguanylic Acid Adjuvant Responses in 1-Year-Old Mice

Next, we determined if tmTNF-Fc (IgG2A) or solTNF-Fc (IgG2A) can enhance CDG adjuvanticity in 1-year-old middle-aged C57BL/6J mice. Again, we used the model antigen NP_6_CGG to separate high- vs. low-affinity antibodies by immunization. We immunized 1-year-old C57BL/6J mice with NP_6_CGG alone, CDG/NP_6_CGG, or CDG/NP_6_CGG with solTNF-Fc or tmTNF-Fc twice at a 2-week interval. Lung memory T cell responses were determined by *ex vivo* recall assay 60 days post the last immunization. Remarkably, both solTNF-Fc and tmTNF-Fc restored CDG-induced lung memory Th1 and Th17 responses in the 1-year-old C57BL/6J mice (Figures 3A,C). The moDC-targeting TNF fusion proteins did not affect lung memory Th2 responses much in the 1-year-old mice (Figure 3B; Figure S2A). Consistently, the moDC-targeting TNF fusion proteins increased lung IgG2c production (Figure S2C). The 1-year-old mice were defective in generating high-affinity antibodies by CDG adjuvant (Figures 3G,K). We found that both solTNF-Fc and tmTNF-Fc enhanced CDG-induced antibodies, especially high-affinity antigen-specific antibodies in the 1-year-old mice (Figures 3G,H,K,L).

Last, we immunized (i.n.) 1-year-old mice with CDG-adjuvanted mucosal pneumococcal protein subunit vaccine CDG/PspA with or without TNF-Fc fusion proteins twice at 2 weeks' interval. Serum anti-PspA IgG was determined 120 days post the last immunization. Remarkably, 1-year-old mice immunized with CDG/PspA plus TNF-Fc fusion proteins had stronger serum anti-PspA IgG 4 months post-immunization than the 1-year-old mice immunized with only CDG/PspA (Figures 3M,N). Together, the data indicated that the moDC-targeting TNF fusion proteins restored the memory Th1, Th17, and high-affinity antibody production in CDG-immunized 1-year-old middle-aged C57BL/6J mice.

Intranasal administration of TNF elicits antibody and Th2 responses (29). However, intranasal administration of solTNF-Fc (IgG2A) or tmTNF-Fc (IgG2A) alone did not induce any antibody or memory Th responses (Figure S4), indicating that the moDC-targeting TNF fusion proteins enhanced CDG adjuvant responses, rather than producing vaccine responses *de novo*. We used a much lower dose of TNF-Fc (IgG2a) (100 ng) than previous TNF (2 μg) used as a mucosal adjuvant (29).

### SolTNF-Fc (IgG2A) and TmTNF-Fc (IgG2A) Enhance 3′,5′-Cyclic Diguanylic Acid-Induced Systemic and Mucosal Antibody Responses in 2-Year-Old C57BL/6J Mice

**Figure 4:**
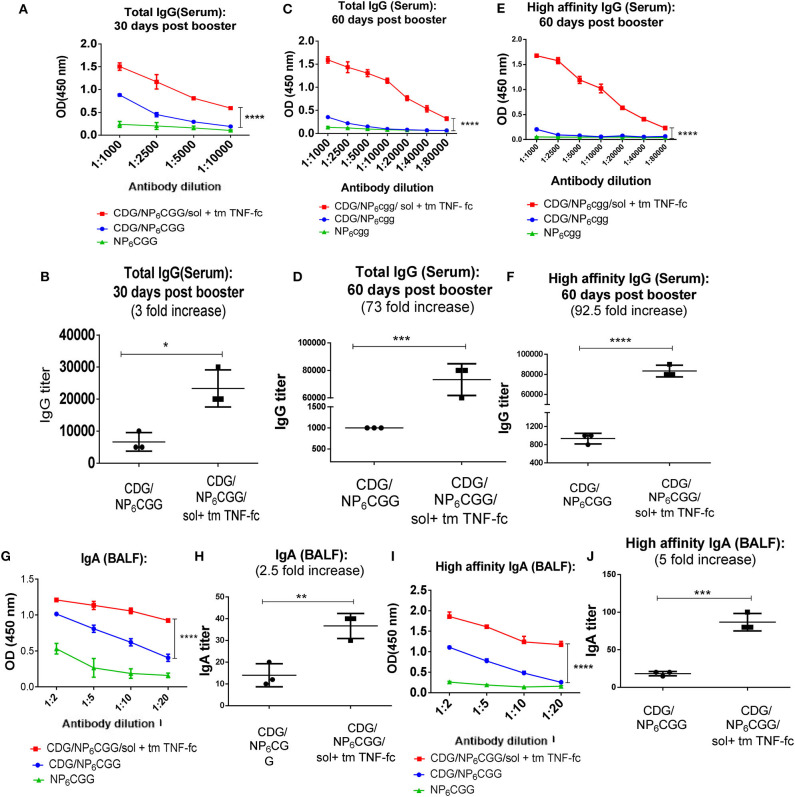
SolTNF-Fc [immunoglobulin (Ig)G2A] and tmTNF-Fc (IgG2A) enhanced 3′,5′-cyclic diguanylic acid (cyclic di-GMP, CDG)-induced humoral response in 2-year-old mice. Two-year-old C57BL/6 mice were intranasally (i.n.) immunized with NP_6_CGG, CDG/NP_6_CGG, or CDG/NP_6_CGG/solTNF-Fc (IgG2A) + tmTNF-Fc (IgG2A) on days 0 and 30. *n* = 4 mice/group. Data are representative of three independent experiments. **(A–F)** Serum samples were collected on 30 and 60 days after the last immunization and total IgG, high-affinity IgG titer were determined by ELISA. **(G–J)** Total IgA, high-affinity IgA in bronchoalveolar lavage fluid (BALF) from immunized mice in **(A)** were determined by ELISA. Antibody endpoint titer was determined by comparing the mice immunized with NP_6_CGG. Significance value was calculated by two-way ANOVA followed by Tukey's multiple comparison test. Error bar represents mean ± SEM. ^*^p < 0.05, ^**^p < 0.01, ^***^p < 0.001, ^****^p < 0.0001.

Next, we used moDC-targeting TNF fusion proteins to restore CDG adjuvanticity in 2-year-old mice that are equal to ~70-year-old humans. We immunized (i.n.) 2-year-old C57BL/6J mice with NP_6_CGG alone, CDG/NP_6_CGG, or CDG/NP_6_CGG with solTNF-Fc (IgG2A) and tmTNF-Fc (IgG2A) twice at a month's interval. Serum antigen-specific antibodies were determined in the serum on days 30 and 60 post last immunization. As expected, CDG did not induce durable and high-affinity IgG in the aged mice (Figures 4A,C,E; Figures S4A–C). In contrast, the addition of solTNF-Fc (IgG2A) and tmTNF-Fc (IgG2A) generated durable serum antigen-specific serum IgG in the 2-year-old mice (Figures 4A–F) (*p* < 0.0001). The enhancement of antibody production was across the spectrum, including augmented production of IgG1, IgG2C by moDC-targeting TNF fusion proteins (Figures S4A,B). We also determined the affinity of the antigen-specific antibody titer in the 2-year-old mice and observed that solTNF-Fc (IgG2A) and tmTNF-Fc (IgG2A) dramatically enhanced (92-folds) CDG-mediated high-affinity antibody production in 2-year-old mice (Figures 4E,F). Last, the enhancement of TNF-Fc fusion proteins was stronger on day 60 (73-folds) than on day 30 (3-folds) post-immunization (Figures 4B,D), suggesting that the TNF-Fc fusion protein increased the durability of serum IgG production in 2-year-old mice.

The induction of antigen-specific IgA response in the lung is highly desirable for preventing respiratory infections such as influenza, *S. pneumococcal*, and SARS-CoV infections. The vast majority of licensed vaccines do not generate lung mucosal IgA responses, even in healthy adults. CDG-adjuvanted vaccines induce antigen-specific lung IgA in the adult but not aged mice (Figure 1C). We next examined if the moDC-targeting TNF fusion proteins restored lung IgA production in the aged mice. We collected BALF from the CDG/NP_6_CGG or CDG/NP6CGG + solTNF-Fc + tmTNF-Fc immunized (i.n.) 2-year-old C57BL/6J mice 60 days post the last immunization and determined antigen-specific IgA titer including high-affinity IgA. A significant elevation in the IgA titer was observed in aged mice immunized with CDG/NP_6_CGG adjuvanted with solTNF-Fc (IgG2A) and tmTNF-Fc (IgG2A) as compared to the mice group immunized with only CDG/NP_6_CGG (*p* < 0.0001) (Figures 4G,H; Figure S4D). Further analysis showed CDG/NP_6_CGG elicited primarily low-affinity lung IgA titer while CDG/NP_6_CGG with moDC-targeting TNF fusion proteins generated high-affinity lung IgA (*p* < 0.0001) in the 2-year-old mice (Figures 4I,J).

Together, the data indicated that the moDC-targeting TNF fusion proteins markedly enhanced CDG-induced durable and high-affinity antibody responses, both in the serum and in the lung, in the 2-year-old C57BL/6J mice.

### SolTNF-Fc (IgG2A) and TmTNF-Fc (IgG2A) Restores Systemic and Lung Memory Th1, Th17 Responses in 3′,5′-Cyclic Diguanylic Acid-Immunized 2-Year-Old C57BL/6J Mice

CDG-induced memory T cell response, primarily Th1 and Th17, is impaired in aged mice (Figure 1D). Vaccine-induced Th1 responses are critical for the protection against intracellular pathogens, while Th17 responses, *via* enhancing neutrophil recruitment, are essential for clearing bacteria, e.g., *Streptococcus pneumoniae* infections. Next, we examined memory T cell responses in 2-year-old mice immunized with the moDC-targeting TNF fusion protein-adjuvanted CDG/NP_6_CGG vaccine 60 days post the last immunization by *ex vivo* recall assay. We examined memory T cell production in the lung (mucosa) and spleen (systemic) in the immunized mice.

**Figure 5:**
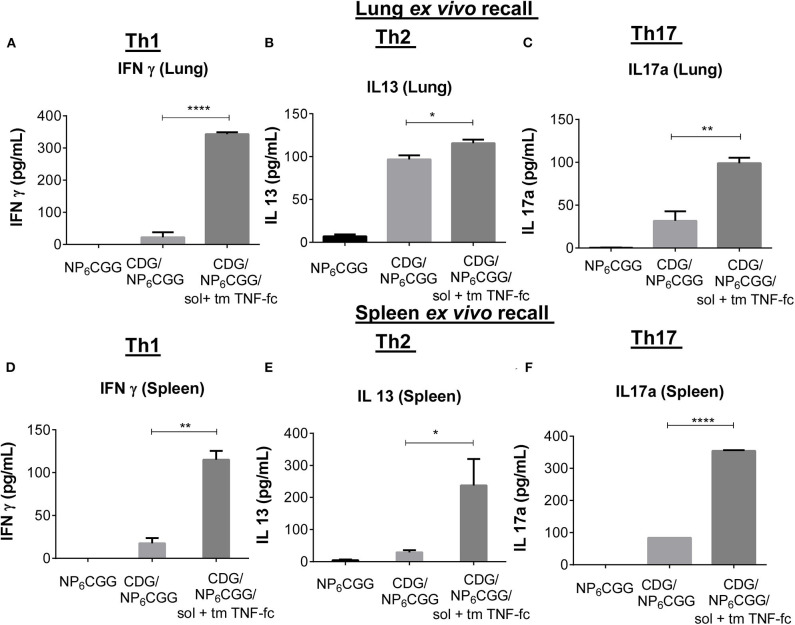
SolTNF-Fc [immunoglobulin (Ig)G2A] and tmTNF-Fc (IgG2A) enhanced 3′,5′-cyclic diguanylic acid (cyclic di-GMP, CDG)-induced memory Th response in 2-year-old mice. Immunized mice (Figure 4) were sacrificed 60 days post-immunization. Splenocytes and lung cells were recalled with 2 μg of NP_6_CGG *ex vivo* for 4 days. Interferon (IFN)-γ, interleukin (IL)-13, and IL-17a levels in the lung **(A–C)** or splenocyte cell culture supernatant **(D–F)** were determined by ELISA. *n* = 4 mice/group. Data are representative of three independent experiments. Significance value was calculated by one- way ANOVA followed by Tukey's multiple comparison test. Error bar represents mean ± SEM. ^*^p < 0.05, ^**^p < 0.01, ^****^p < 0.0001.

As expected, no Th1 (IFN-γ) or Th17 (IL-17a) was detected in the lungs of aged mice immunized with CDG/NP_6_CGG only (Figures 5A,C). Similar to the 1-year-old mice, Th2 (IL-13), and IL-5 response was detected in the lungs from immunized aged mice (Figure 5B; Figure S6B). Remarkably, aged mice immunized with CDG/NP_6_CGG plus moDC-targeting TNF fusion proteins elicited a significantly high level of Th1 (*p* < 0.0001), Th17 (*p* < 0.01) responses in the lungs (Figures 5A,C). Lung memory Th2 responses were not affected much by the addition of TNF-Fc fusion proteins (Figure 5B; Figure S6B). Similarly, spleen memory Th1 and Th17 responses were enhanced in CDG/NP_6_CGG/TNF-Fc immunized 2-year-old mice (Figures 5D,F). Different from the lung, CDG/NP_6_CGG did not induce spleen memory Th2 responses in 2-year-old mice (Figure 5E; Figure S6A). However, the addition of TNF-Fc fusion proteins enhanced CDG/NP_6_CGG-induced spleen memory Th2 responses (Figure 5E; Figure S6A). The data, thus, indicated that solTNF-Fc (IgG2A) and tmTNF-Fc (IgG2A) restored CDG-induced memory T cell response in the systemic as well as mucosal compartments in the aged C57BL/6J mice.

## Discussion

In this report, we take a mechanism-guided rationally designed approach to improve vaccine efficacy in the middle-aged and aged mice. Our vaccine strategy is (i) to identify the *in vivo* mode of action of CDG adjuvant in the adult mice (20, 22); (ii) to define the defects in the aged mice that compromises CDG adjuvanticity *in vivo*; (iii) to design new biologics to circumvent the defective component in CDG mode of action and restore CDG adjuvanticity in the aged mice. Our new vaccine strategy focuses on moDC targeting and TNF induction because (i) CDG adjuvanticity is mediated by two DC subsets: cDC2 and moDCs (22); (ii) CDG-induced cDC2 production of TNF is critical for CDG adjuvanticity (19, 20, 22); (iii) aged mice had decreased cDC2 but increased moDCs; and (iv) CDG did not induce lung TNF in the aged mice. We designed the TNF-Fc (IgG2A) to targeted activating moDCs by TNF. Our strategy successfully restored CDG adjuvanticity in the middle-aged and aged mice.

Aging leads to a progressive decline of the innate and adaptive immune system that likely causes the reduced vaccine efficacy in the aged. Our vaccine strategy intends to adapt rather than change or reverse the immunosenescence in the aged. The current vaccine strategies to enhance vaccine responses in the elderly are empirical due to our limited understanding of immunosenescence (30). The success of our targeted vaccine strategy depends on the knowledge of CDG adjuvant mode of action in the adult and aged mice. This new strategy does not require a deep understanding of immunosenescence; thus, it is likely more practical for the development of vaccines for the aged.

It remains to be determined if our moDC-targeting TNF fusion proteins will enhance non-CDN vaccines in the aged. Most vaccines likely will have a mode of action different from CDNs. To adapt this targeted vaccine strategy requires efforts to understand the mode of action for each vaccine, which may be challenging. To solve this limitation, we are exploring if our moDC-targeting TNF fusion proteins can enhance the efficacy of non-CDN vaccines in the aged.

CDNs are excellent adjuvants eliciting not only long-lasting and potent antibody responses but also Th1, Th2, Th17, and antitumor CD8 T cell response. There were tremendous interests in translating CDNs into the clinic. For example, in 2015, Aduro Biotech and Novartis announced a $250M-plus initiative to develop CDNs as cancer immunotherapies (31). Surprisingly, how aging affects CDN vaccine adjuvanticity was not well-addressed.

In early 2019, Darling et al. (32) showed that CDNs induced serum antibody production in 20-month-old female BALB/c mice. They did not examine memory T cell responses or distinguish high- and low-affinity antibody production (32). They immunized mice [subcutaneously (s.c.)] with 50 μg OVA antigen and 20 μg CDNs (32), a rather high dose of CDNs. In late December 2019, Vassilieva et al. (33) showed that 2′3′-cGAMP (5 μg) adjuvanted with 1 μg of hemagglutinin (HA) [intradermally (i.d.)] did not induce antibodies or protective immunity in the 19-month-old female BALB/c mice. And 100% aged mice immunized (i.d.) with 2′3′-cGAMP/HA died subsequently from influenza infection (33). In comparison, 75% of aged mice immunized with Quil-A, a saponin adjuvant, with HA antigen immunization (i.d.) survived from influenza infection (33). Our observations here are in line with this latest result. We found that the antigen-specific high-affinity antibody production and memory Th1, Th17 responses were markedly reduced in 2-year-old mice. Our results further showed that even 1-year-old mice were defective in CDG-induced Th1 and Th17 responses.

Vaccination offers the most efficient and cost-effective method of preventing infectious diseases. However, vaccines are less effective in the elderly. For instance, the influenza vaccine has an efficacy between 70 and 90% in children and adults but dropping to 30–50% for those over 65 years of age (4, 34). Similarly, responses to pneumococcal polysaccharide and hepatitis B vaccines are compromised by the old age (35). In addition, ~83% of pneumonia deaths in the US occurred in the elderly (36, 37). Last, people 45 and older (middle-aged and aged) account for ~97% of COVID-19 deaths (1). Methods to improve vaccine protection in the elderly are highly desirable. We used the model antigen NP_6_CGG to reveal a defect of high-affinity antibody and Th1/17 induction in the middle-aged and aged mice. We observed the aged-related defect using H1N1-NP and PspA antigens. TNF-Fc fusion protein enhanced the duration (120 days post-immunization) and the magnitude of CDG/PspA antibody production in 1-year-old mice. CDN-adjuvanted protein subunit vaccines protected adult mice from respiratory infections such as influenza (38, 39), bacterial pneumonia (40), *Mycobacterium tuberculosis* (41), and anthrax (42). CDNs were also therapeutic for cancer in adult mice (13, 14). Future studies are needed to determine if the TNF-Fc fusion proteins can improve protection in CDN-adjuvanted vaccines for infectious diseases and cancers in the elderly.

As a vaccine specific to the elderly, safety is paramount. Intranasally administered CDG (5 μg/mouse) does not cause acute toxicity in mice (20, 43, 44) and is proven safe in various animal models (38, 40, 44–47). Clinical trials on CDNs (ClinicalTrials.gov NCT02675439, NCT03010176, NCT03172936, NCT03937141, and NCT0414414) have established an excellent safety file in humans, including the elderly. The human body makes TNF. Low-dose (ng) targeted delivered TNF biologics are likely safe in the elderly as well. Nevertheless, a future study is needed to establish a safety profile for the CDG/TNF biologics vaccines for the elderly.

Last, the moDC-targeting TNF fusion proteins applied here did not have adjuvant activity when administered in the absence of CDG. Furthermore, TNF when used as a mucosal adjuvant mainly induces Th2 responses (29). Here, moDC-targeting TNF biologics enhanced memory Th1 and Th17-immunity of the CDG. We propose that TNF fusion proteins enhanced CDG adjuvanticity rather than acted as a separate adjuvant to replace CDG in aged mice. In all, we showed that aging negatively influences CDG adjuvanticity, especially CDG-induced memory Th1 and Th17 responses. A new moDC-targeting strategy can restore CDG adjuvanticity in middle-aged and aged mice.

## Data Availability Statement

All datasets presented in this study are included in the article/Supplementary Material.

## Ethics Statement

The animal study was reviewed and approved by Institutional Animal Care and Use Committee, University of Florida.

## Author Contributions

SM and LJ conceived the research. LJ designed the experiments, wrote the manuscript, and supervised the research. HG, SM, DK, and LJ performed experiments and analyzed the data. HG drafted the manuscript. All authors contributed to the article and approved the submitted version.

## Conflict of Interest

LJ and SM are co-Inventors on a patent (PCT/US19/53548) on the moDCs-targeting TNF fusion proteins. The remaining authors declare that the research was conducted in the absence of any commercial or financial relationships that could be construed as a potential conflict of interest.
