# Supplementary material for: New MoDC-Targeting TNF Fusion Proteins Enhance Cyclic Di-GMP Vaccine Adjuvanticity in Middle-Aged and Aged Mice
Source: Front Immunol. 2020 Aug 7;11:1674. doi: 10.3389/fimmu.2020.01674 (PMC7427090; doi:10.3389/fimmu.2020.01674)
Supplement: Supplementary file 1 [file Data_Sheet_1.PDF]

Supplementary Material

1.1 Supplementary Figures

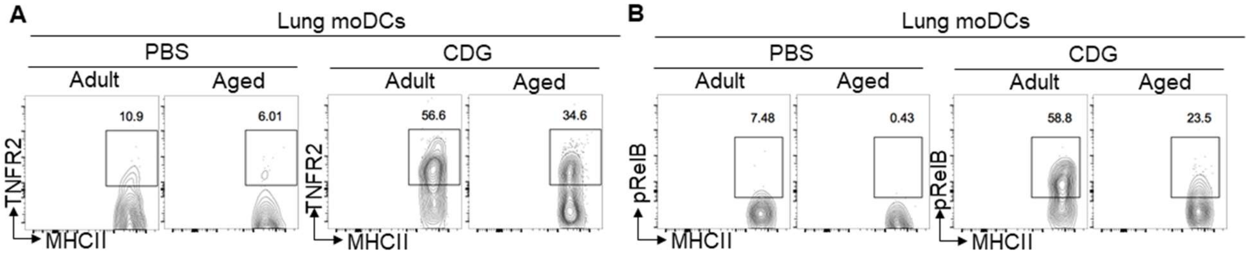

Supplementary Figure 1. CDG mediated lung moDC activation is reduced in aged mice. (**A, B**) Flow cytometry analysis of TNFR2 and pRelB expression in lung moDC of adult and aged C57BL/6 mice treated (*i.n.*) with PBS or CDG for 16 h as in Figure 2D, 2E. n=4mice/group. Data are representative of three independent experiments.

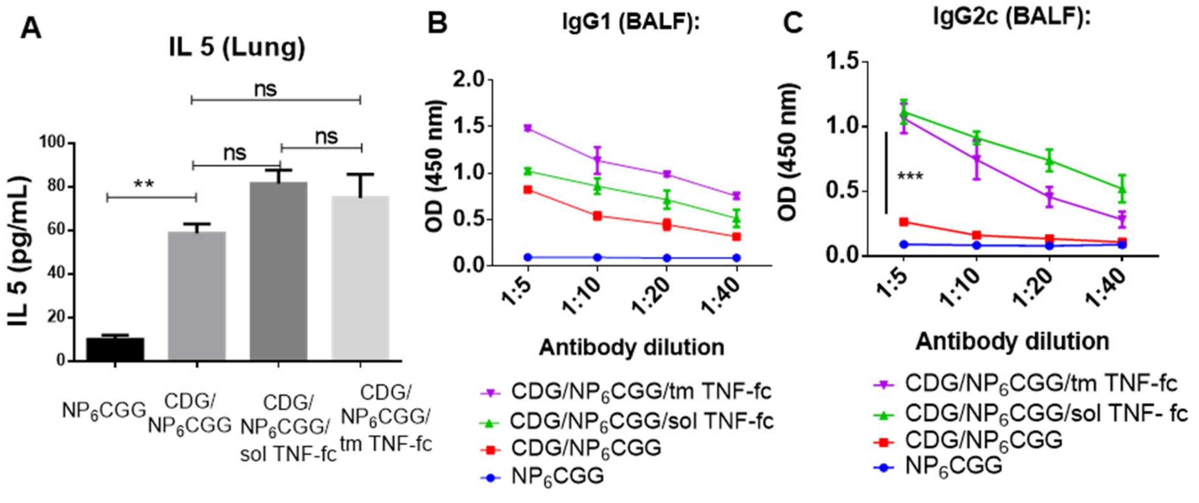

Supplementary Figure 2. solTNF-Fc (IgG2A) and tmTNF-Fc (IgG2A) enhanced CDG mediated humoral antibody response in 1-year-old mice: (A) 1-year-old C57BL/6 mice were immunized (*i.n.*) twice with NP$_6$CGG, CDG/NP$_6$CGG, CDG/NP$_6$CGG/solTNF-Fc or CDG/NP$_6$CGG/tmTNF-Fc on day 0 and 14 as in Figure 3. Lung cells from immunized mice were harvested 60 days post last immunization and recalled *ex vivo* with NP$_6$CGG (2 µg) for 4 days. IL-5 cytokine was determined in culture supernatant by ELISA. n=4mice/group. Data are representative of three independent experiments. **(B-C)** BALF samples from mice in (**A**) were collected 60 days post immunization and analyzed for NP-specific antibody titres by ELISA. Data are representative of three independent experiments. Significance value was calculated by one- way ANOVA followed by Tukey's multiple comparison test (**A**) or two- way ANOVA followed by Tukey's multiple comparison test (**B-C**). Error bar represents mean ± SEM. **p<0.01. ***p< 0.001, n.s: not significant.

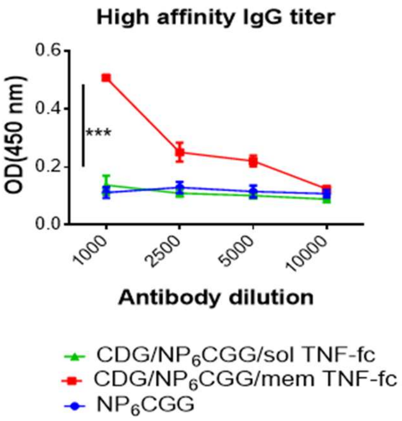

Supplementary Figure 3. tmTNF-Fc(IgG2A) enhanced CDG-induced IgG response in the IRF4$^{fl/fl}$CD11c^{cre} mice. IRF4$^{fl/fl}$ CD11c ^{cre} mice were immunized intranasally with either NP_6CGG or CDG/NP_6CGG/sol TNF-Fc(IgG2A) or CDG/NP_6CGG/tmTNF-Fc(IgG2A). NP-specific IgG titres in the serum was determined by ELISA after 14 days of immunization. n=4mice/group. Significance value was calculated by two- way ANOVA followed by Tukey's multiple comparison test. Error bar represents mean ± SEM. ***p<0.001

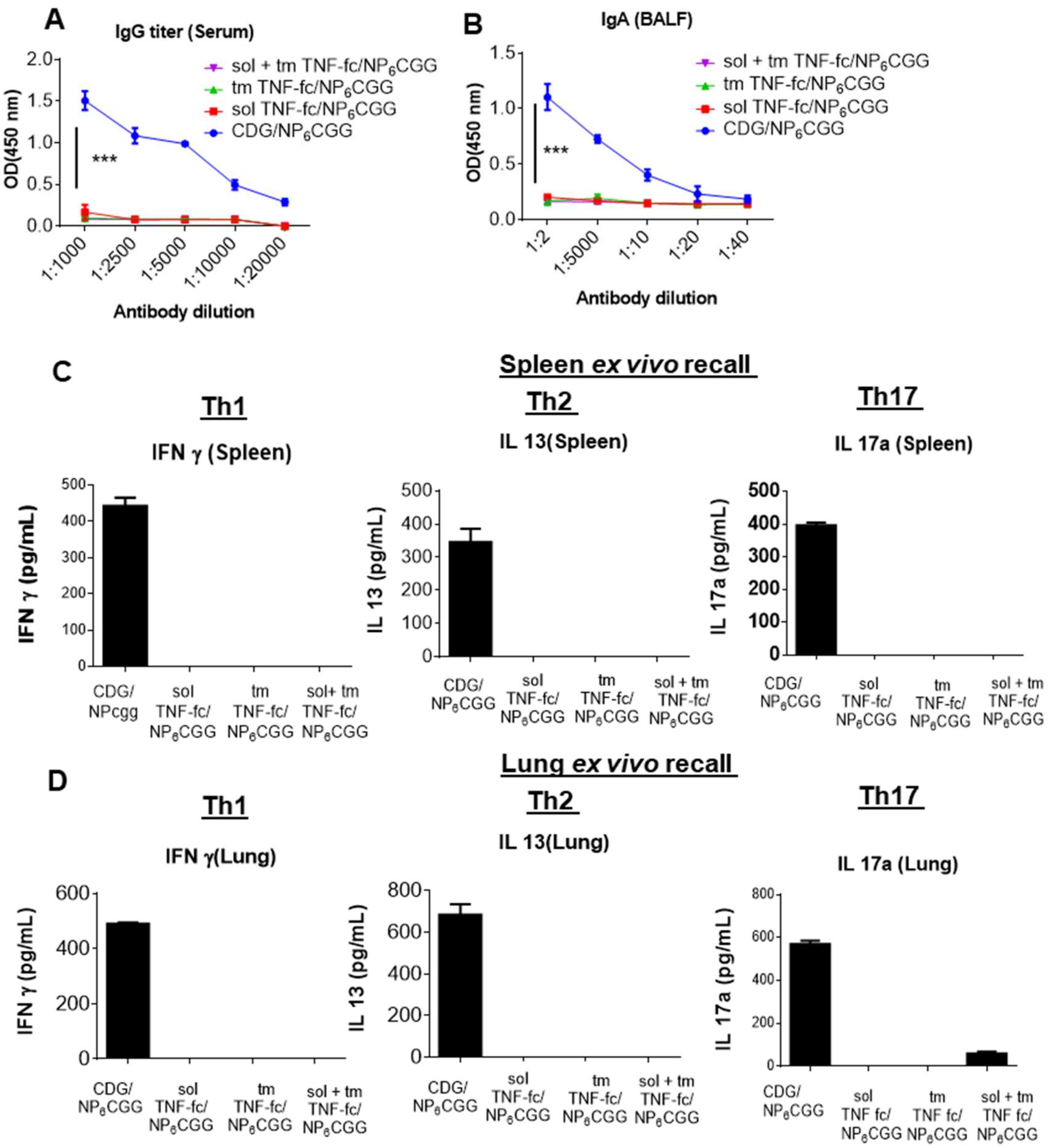

Supplementary Figure 4. solTNF-Fc(IgG2A) or tmTNF-Fc(IgG2A) alone do not induce humoral or memory Th response. 3 month old C57BL/6 mice were immunized with either CDG/NP$_6$CGG, solTNF-Fc(IgG2A)/NP$_6$CGG, tmTNF-Fc(IgG2A)/NP$_6$CGG or solTNF-Fc(IgG2A) + tmTNF-Fc(IgG2A)/NP$_6$CGG on day 0 and 14. **(A,B)** Serum and **BALF** samples were collected on day 30 after immunization and analyzed for IgG and IgA titer by ELISA. Splenocytes and lung cells were recalled with NP$_6$CGG (2μg) *ex vivo* for 4 days. **(C)** Splenocyte and **(D)** lung cell culture supernatants were analyzed for IFN γ, IL 13 and IL 17a levels by ELISA. n=4mice/group. Data are representative of three independent experiments. Significance value was calculated by two- way ANOVA followed by Tukey's multiple comparison test. Error bar represents mean ± SEM. ***p<0.001.

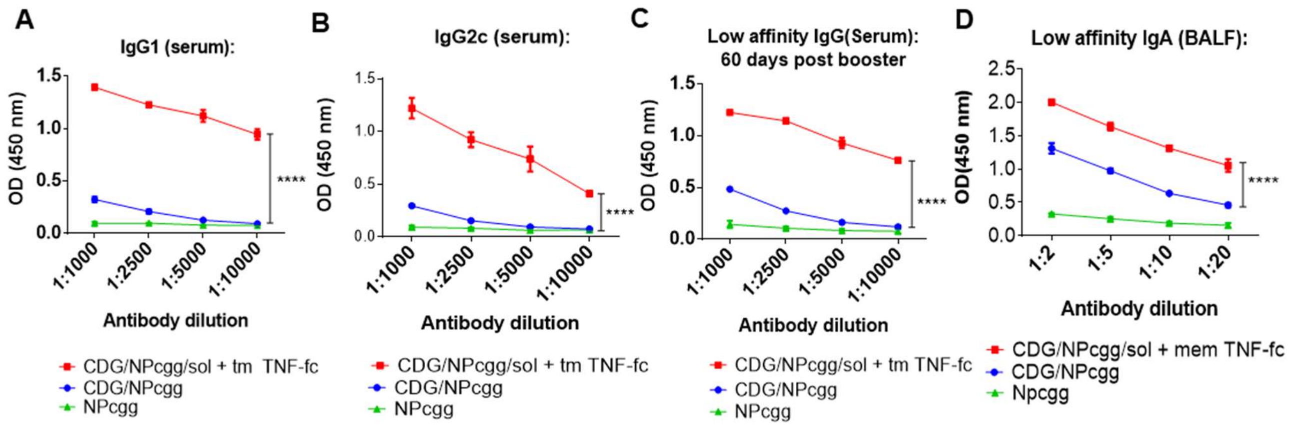

Supplementary Figure 5. solTNF-Fc (IgG2A) and tmTNF-Fc (IgG2A) enhanced CDG-mediated systemic and mucosal antibody production in 2 -year-old mice. 2- year- old C57BL/6 mice were *i.n.* immunized with either NP_6CGG, CDG/NP_6CGG or CDG/NP_6CGG/solTNF-Fc (IgG2A) + tmTNF-Fc (IgG2A) on day 0 and day 14. **(A- C)** IgG1, IgG2c and low affinity IgG levels in the serum were determined by ELISA 60 days post last immunization**. (D)** Low affinity IgA level was determined in the BALF 60 days post immunization by ELISA. n=4mice/group. Data are representative of three independent experiments. Significance value was calculated by two- way ANOVA followed by Tukey's multiple comparison test. Error bar represents mean ± SEM. ****p<0.0001.

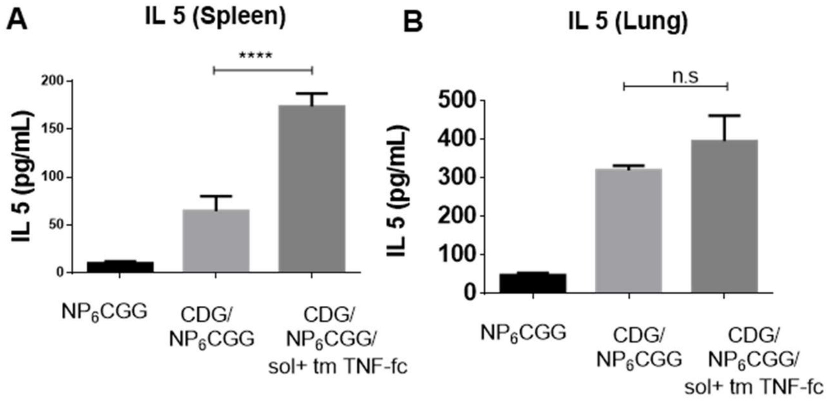

Supplementary Figure 6. solTNF-Fc(IgG2A) and tmTNF-Fc(IgG2A) do not enhance lung memory Th2 response in 2-year-old mice. (A-B) Immunized 2-year-old mice (**Figure 4**) were sacrificed 60 days post immunization. Splenocytes and lung cells were recalled with 2 µg of NP_6CGG *ex vivo* for 4 days. IL-5 in culture supernatant were determined by ELISA. n=4mice/group. Data are representative of three independent experiments. Significance value was calculated by one-way ANOVA followed by Tukey's multiple comparison test. Error bar represents mean ± SEM. ****p<0.0001. n.s, not significant.
